# Supplementary material for: A landscape of gene expression regulation for synovium in arthritis
Source: Nat Commun. 2024 Feb 15;15:1409. doi: 10.1038/s41467-024-45652-x (PMC10869817; doi:10.1038/s41467-024-45652-x)
Supplement: Supplementary file 1 — Supplementary Information [file 41467_2024_45652_MOESM1_ESM.pdf]

Supplementary Information

Supplementary Tables
Supplementary Figures

Supplementary Tables

Supplementary Table 1. Summary information of patients including quality control.
Supplementary Table 2. Datasets used in epigenetic annotation for independent SNPs.
Supplementary Table 3. Functional enrichments of top 10 enriched TFs.
Supplementary Table 4. Previous GWAS studies used to collect known colocalized genes.
Supplementary Table 5. GEO datasets utilized in differential expression analysis.
Supplementary Table 6. A list of all primers that using in this study.
Supplementary Table 7. Epigenetic data for eQTac analysis.

Supplementary Table 1. Summary information of patients including quality control.

	Disease	Osteoarthritis		Rheumatoid arthritis
	Analysis	eQTL	ATAC-seq	ATAC-seq
	Patients counts	245	3	7
Data availability	Genotype available	245	-	-
	RNA-seq available	210	-	-
QC exclusions	Low expression correlation	6	-	-
	Heterozygosity rate outliers	2	-	-
	Lack of RNA	35	-	-
Sample information	Age (range)	46-84	-	-
	Age (mean)	67	-	-
	Sex ratio (Female:Male)	168:77	-	-

Supplementary Table 2. Datasets used in epigenetic annotation for independent SNPs.

		*Available datasets								
**Label names**	**Samples description**	**ATAC-seq**	**H3k4me1**	**H3k4me3**	**H3K27ac**	**H3K36me3**	**H3K27me3**	**H3K9me3**	**Download links**	**Pubmed ID**
GSE163548_88.healthy.KN	Unstimulated primary synovium FLS from healthy knee joint		*	*	*	*	*	*	https://www.ncbi.nlm.nih.gov/geo/download/?acc=GSE163548&format=file	34433485[1]
GSE163548_384.OA.SH	Unstimulated primary synovium FLS from OA shoulder joint		*	*	*	*	*	*	https://www.ncbi.nlm.nih.gov/geo/download/?acc=GSE163548&format=file	34433485[1]
GSE163548_343.OA.MCP	Unstimulated primary synovium FLS from OA metacarpophalangeal joint		*	*	*	*	*	*	https://www.ncbi.nlm.nih.gov/geo/download/?acc=GSE163548&format=file	34433485[1]
GSE163548_335.OA.KN	Unstimulated primary synovium FLS from OA knee joint		*	*	*	*	*	*	https://www.ncbi.nlm.nih.gov/geo/download/?acc=GSE163548&format=file	34433485[1]
GSE112655_OA.11	OA patient Fibroblast-like	*	*	*	*	*	*	*	https://www.ncbi.nlm.nih.gov/geo/query/acc.cgi?acc=GSE112655	29765031[2]
GSE112655_OA.10	OA patient Fibroblast-like	*	*	*	*	*	*	*	https://www.ncbi.nlm.nih.gov/geo/query/acc.cgi?acc=GSE112655	29765031[2]
GSE112655_OA.09	OA patient Fibroblast-like	*	*	*	*	*	*	*	https://www.ncbi.nlm.nih.gov/geo/query/acc.cgi?acc=GSE112655	29765031[2]
GSE112655_OA.08	OA patient Fibroblast-like	*	*	*	*	*	*	*	https://www.ncbi.nlm.nih.gov/geo/query/acc.cgi?acc=GSE112655	29765031[2]
GSE112655_OA.07	OA patient Fibroblast-like	*	*	*	*	*	*	*	https://www.ncbi.nlm.nih.gov/geo/query/acc.cgi?acc=GSE112655	29765031[2]
GSE112655_OA.06	OA patient Fibroblast-like	*	*	*	*	*	*	*	https://www.ncbi.nlm.nih.gov/geo/query/acc.cgi?acc=GSE112655	29765031[2]
GSE112655_OA.05	OA patient Fibroblast-like	*	*	*	*	*	*	*	https://www.ncbi.nlm.nih.gov/geo/query/acc.cgi?acc=GSE112655	29765031[2]
GSE112655_OA.04	OA patient Fibroblast-like	*	*	*	*	*	*	*	https://www.ncbi.nlm.nih.gov/geo/query/acc.cgi?acc=GSE112655	29765031[2]
GSE112655_OA.03	OA patient Fibroblast-like	*	*	*	*	*	*	*	https://www.ncbi.nlm.nih.gov/geo/query/acc.cgi?acc=GSE112655	29765031[2]
GSE112655_OA.02	OA patient Fibroblast-like	*	*	*	*	*	*	*	https://www.ncbi.nlm.nih.gov/geo/query/acc.cgi?acc=GSE112655	29765031[2]
GSE112655_OA.01	OA patient Fibroblast-like	*	*	*	*	*	*	*	https://www.ncbi.nlm.nih.gov/geo/query/acc.cgi?acc=GSE112655	29765031[2]
ARD2020_OA.ns	Unstimulated synovial fibroblasts from OA patients		*	*	*				https://humandbs.biosciencedbc.jp/files/hum0207/hum0207.v1.ChIP.v1.zip	33139312[3]

Note: "" indicates the available datasets for this epigenomic marker. Blank indicates the not availabel datasets.

Supplementary Table 3. Functional enrichments of top 10 enriched TFs.

Term ID	Term description	Observed gene count	Background gene count	Strength (Log10(observed / expected))	False discovery rate	Matching proteins (labels)
GO:0033152	Immunoglobulin V(D)J recombination	2	8	2.69	0.0036	YY1,TCF3
GO:0032688	Negative regulation of interferon-beta production	3	13	2.66	0.00013	RELB,YY1,REL
GO:0038061	NIK/NF-kappaB signaling	2	36	2.04	0.0429	RELB,REL
GO:0030183	B cell differentiation	3	129	1.66	0.0103	YY1,TCF3,PAX5
GO:0030098	Lymphocyte differentiation	4	279	1.45	0.0029	RELB,YY1,TCF3,PAX5

Supplementary Table 4. Previous GWAS studies used to collect known colocalized genes.

Trait	Article	Pubmed ID	eQTL tissues
OA	Deciphering osteoarthritis genetics across 826,690 individuals from 9 populations.	34822786[4]	GTEx tissues
OA	Genome-wide analyses using UK Biobank data provide insights into the genetic architecture of	29559693[5]	GTEx tissues
OA	Identification of new therapeutic targets for osteoarthritis through genome-wide analyses of UK Biobank	30664745[6]	GTEx tissues
RA	Genetics of rheumatoid arthritis contributes to biology and drug discovery.	24390342[7]	Monocytes, PBMCs, T cells
RA	Large-scale genome-wide association study in a Japanese population identifies novel susceptibility loci across different diseases.	32514122[8]	GTEx tissues
RA	Large-scale meta-analysis across East Asian and European populations updated genetic architecture and variant-driven biology of rheumatoid arthritis, identifying 11 novel susceptibility loci.	33310728[9]	GTEx tissues, DICE
RA	Multi-ancestry genome-wide association analyses identify novel genetic mechanisms in rheumatoid	32514122[8]	GTEx tissues, Monocytes, Neutrophils, T
AS	Analysis of five chronic inflammatory diseases identifies 27 new associations and highlights disease-specific patterns at shared loci.	26974007[10]	Peripheral blood
JIA	Dense genotyping of immune-related disease regions identifies 14 new susceptibility loci for juvenile idiopathic arthritis.	23603761[11]	LCLs, T cells, Fibroblast

Supplementary Table 5. GEO datasets utilized in differential expression analysis.

Trait	GEO number	Case	Control	Case count	Control count	Pubmed id	Notes	Method
OA	GSE46750	OA patients synovial membrane inflammatory	OA pateints synovial membrane normal/reactive	12	12	24757147[12]		GEO2R (limma)
OA	GSE12021	OA patients synovium	normal control synovium	10	4	18721452[13]	GPL97	GEO2R (limma)
OA	GSE12021	OA patients synovium	normal control synovium	10	9	18721452[13]	GPL96	GEO2R (limma)
OA	GSE55235	OA patients synovium	normal control synovium	10	10	24690414[14]		GEO2R (limma)
OA	GSE55457	OA patients synovium	normal control synovium	10	10	24690414[14]		GEO2R (limma)
OA	GSE82107	OA patients synovium	normal control synovium	10	7	27870898[15]		GEO2R (limma)
RA	GSE12021	RA patients synovium	normal control synovium	12	9	18721452[13]	GPL96	GEO2R (limma)
RA	GSE12021	RA patients synovium	normal control synovium	12	4	18721452[13]	GPL97	GEO2R (limma)
RA	GSE55235	RA patients synovium	normal control synovium	10	10	24690414[14]		GEO2R (limma)
RA	GSE55457	RA patients synovium	normal control synovium	13	10	24690414[14]		GEO2R (limma)
AS	GSE41038	Ankylosing spondylitis pateints synovium	normal control synovium	2	4	24330574[16]		GEO2R (limma)
JIA	GSE165626	JIA FLS cultured	normal FLS cultured	9	9	33980237[17]		GEO2R (limma)

Supplementary Table 6. A list of all primers that using in this study.

Name	Sequence(5'->3')
rs142845557-genotyping-F	TCATGCAGGGACTCTCAAGC
rs142845557-genotyping-R	GCACAGGGGTGGGATGAAAA
rs142845557-KO-sgRNA1-F	ACCGCAAGGCGTGGGGGTAATATC
rs142845557-KO-sgRNA1-R	TAACGATATTACCCCCACGCCTTG
rs142845557-KO-sgRNA2-F	ACCGTGGCACAATAAGGGAACCTC
rs142845557-KO-sgRNA3-R	TAACGAGGTTCCCTTATTGTGCCA
JAZF1 -qPCR-F	AGCACATCGAGGACAACCAC
JAZF1 -qPCR-R	TTAGGGACTCCTGCTCTCGG
GAPDH -qPCR-F	GGAGCGAGATCCCTCCAAAAT
GAPDH -qPCR-R	GGCTGTTGTCATACTTCTCATGG

Supplementary Table 7. Epigenetic data for eQTac analysis.

Datasets	Tissues	Description	Article	Pubmed ID	Download link
ABC model prediction	131 tissues	ABC predictions in 131 cell types and tissues (all element-gene connections with ABC scores >= 0.015	Genome-wide enhancer maps link risk variants to disease genes	33828297[18]	ftp://ftp.broadinstitute.org/outgoing/lincRNA/ABC/AllPredictions.AvgHiC.ABC0.015.minus150.ForABCPaperV3.t
EpiMap gene-enhancer link	31 tissues	Four sets of gene-enhancer links based on expression-enhancer activity correlation, mark activity, and distance across 304 matched RNA-seq and epigenomic datasets, then aggregated for each sample group in 31 tissues (Adipose, Brain, etc.)	Regulatory genomic circuitry of human disease loci by integrative epigenomics	33536621[19]	https://personal.broadinstitute.org/cboix/epimap/links/pergroup/
EpiMap HMM18	31 tissues	Chromatin state calls using ChromHMM , calculated using the 18-state Roadmap model	Regulatory genomic circuitry of human disease loci by integrative epigenomics	33536621[19]	https://personal.broadinstitute.org/cboix/epimap/ChromHMM/observed_aux_18_hg19/CALLS/
synovium.nonstim_loops	Synovium	Called loop in synovial fibroblasts under unstimulated condition	Parsing multiomics landscape of activated synovial fibroblasts highlights drug targets linked to genetic risk of rheumatoid arthritis	33139312[3]	https://humandbs.biosciencedbc.jp/files/hum0207/hum0207.v1.HiC.v1.zip
synovium.8mix_loops	Synovium	Called loop in synovial fibroblasts under the mixture of 8 cytokines (IFN-α, IFN-γ, TNF-α, IL-1β, IL-6/sIL-6R, IL-17, TGF-β1, IL-1) stimulated condition	Parsing multiomics landscape of activated synovial fibroblasts highlights drug targets linked to genetic risk of rheumatoid arthritis	33139312[3]	https://humandbs.biosciencedbc.jp/files/hum0207/hum0207.v1.HiC.v1.zip
synovium.TNFa_loops	Synovium	Called loop in synovial fibroblasts under TNF-α stimulated condition	Parsing multiomics landscape of activated synovial fibroblasts highlights drug targets linked to genetic risk of rheumatoid arthritis	33139312[3]	https://humandbs.biosciencedbc.jp/files/hum0207/hum0207.v1.HiC.v1.zip
synovium.Stim_cHiC	Synovium	Capture Hi-C in primary synovium FLS under TNF stimulated condition	Functional genomics atlas of synovial fibroblasts defining rheumatoid arthritis heritability	34433485[1]	https://ftp.ncbi.nlm.nih.gov/geo/series/GSE163nnn/GSE163548/suppl/GSE163548_Stim_Chicago_washU_text.txt.
synovium.UnStim_cHiC	Synovium	Capture Hi-C in primary synovium FLS under unstimulated condition	Functional genomics atlas of synovial fibroblasts defining rheumatoid arthritis heritability	34433485[1]	https://ftp.ncbi.nlm.nih.gov/geo/series/GSE163nnn/GSE163548/suppl/GSE163548_Unstim_Chicago_washU_text.

Supplementary Figures

Supplementary Fig. 1. The overlap between our eQTL results and the previous synovium eQTL study (Steinberg et.al[20]).

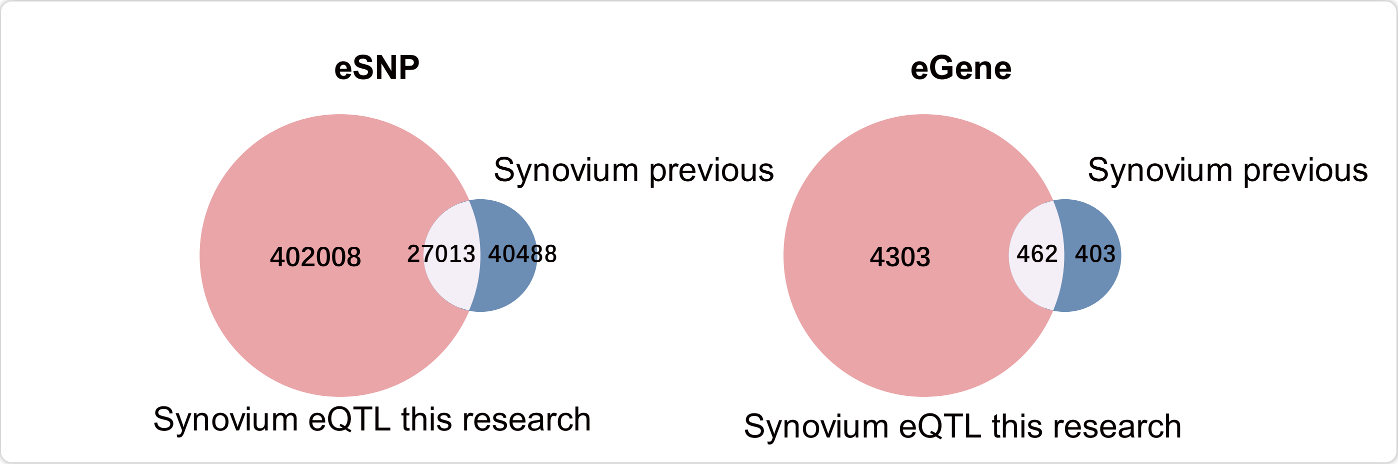

Supplementary Fig. 2. The distribution of eGene count per eSNP.

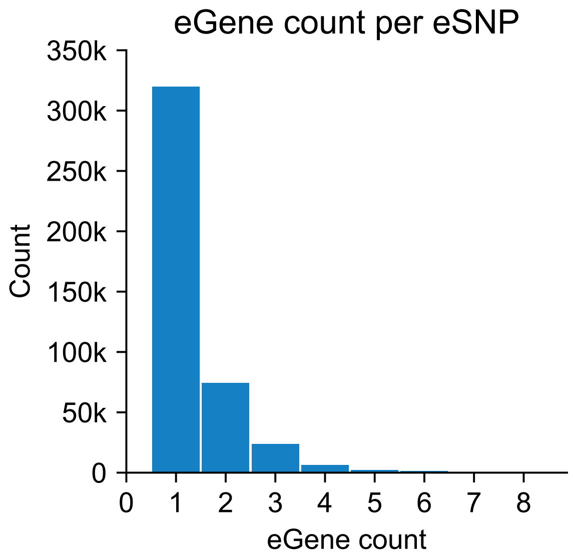

Supplementary Fig. 3. Density scatterplot showed the correlation between top SNP distance and the top eQTL effects. Distances and effect sizes were log transformed. The colors of points indicate the density of distribution.

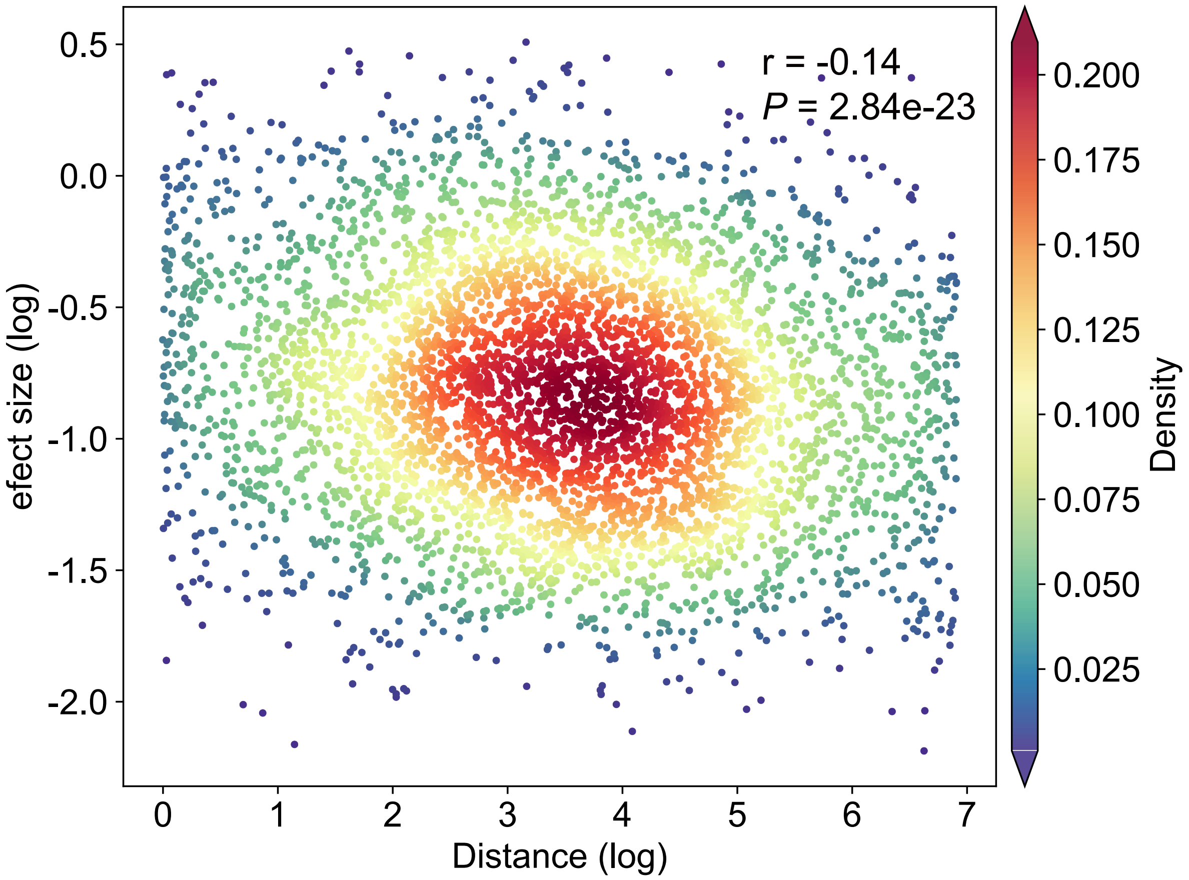

Supplementary Fig. 4. Density scatterplot showed the correlation between top eQTL effect sizes and the target gene expression CVs (coefficients of variation). CVs were adjusted by gene expression. Effect sizes and expression CVs were log transformed. The colors of points indicate the density of distribution.

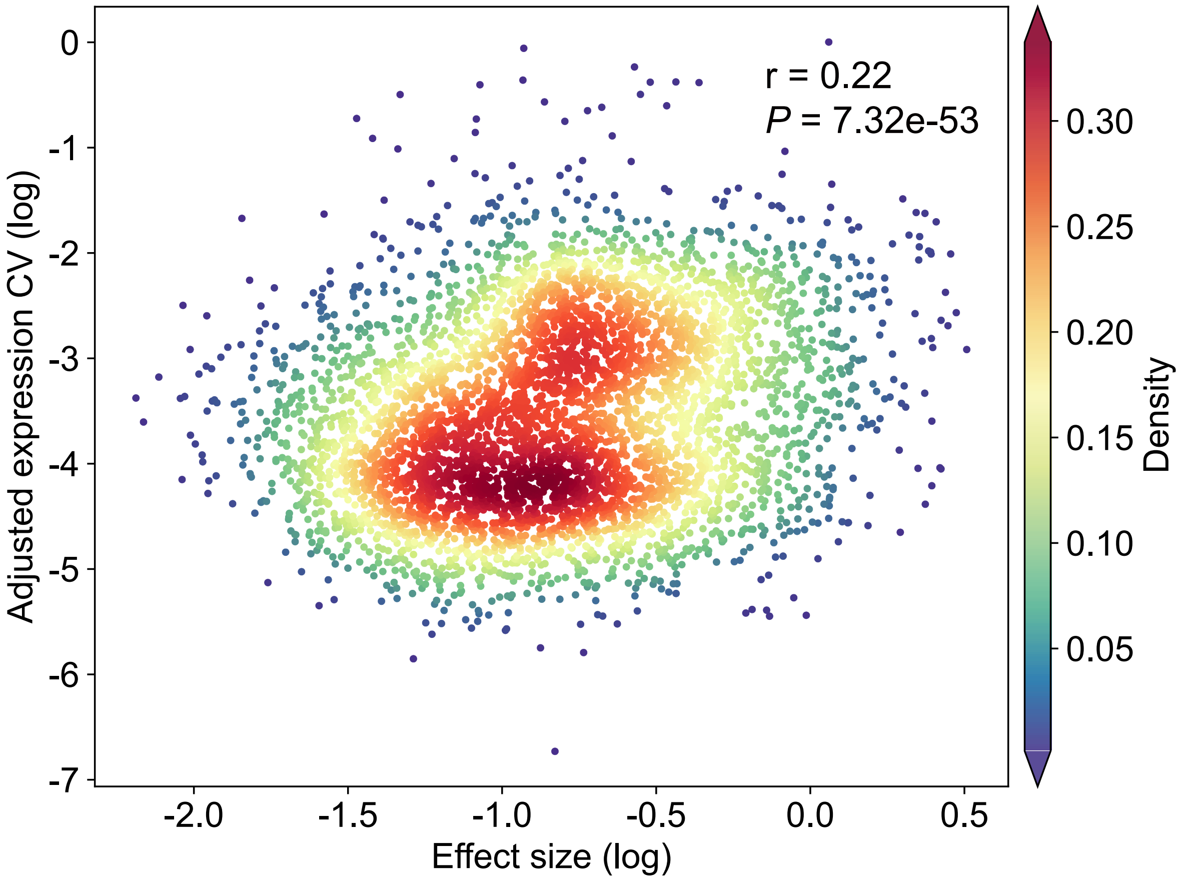

Supplementary Fig. 5. The distance of primary signals and secondary signals to target gene TSS, in which the signals located on target gene body. (a). Distance to target gene TSS. (b) Distance to target gene TSS normalized by gene length. N = 5380 for independent eQTLs in (a) and (b) . The boxplots represent 25th, 50th (median), and 75th percentiles, and whiskers extend to 1.5 times the interquartile range. The green rhombuses represent the mean of each box. ns: $p > 0.05$; *: $p \leq 0.05$; **: $p \leq 0.01$; ***: $p \leq 0.001$; ****: $p \leq 0.0001$. All the statistical tests in (a)-(b) are two-sided Mann-Whitney test, no adjustments were made for multiple comparisons.

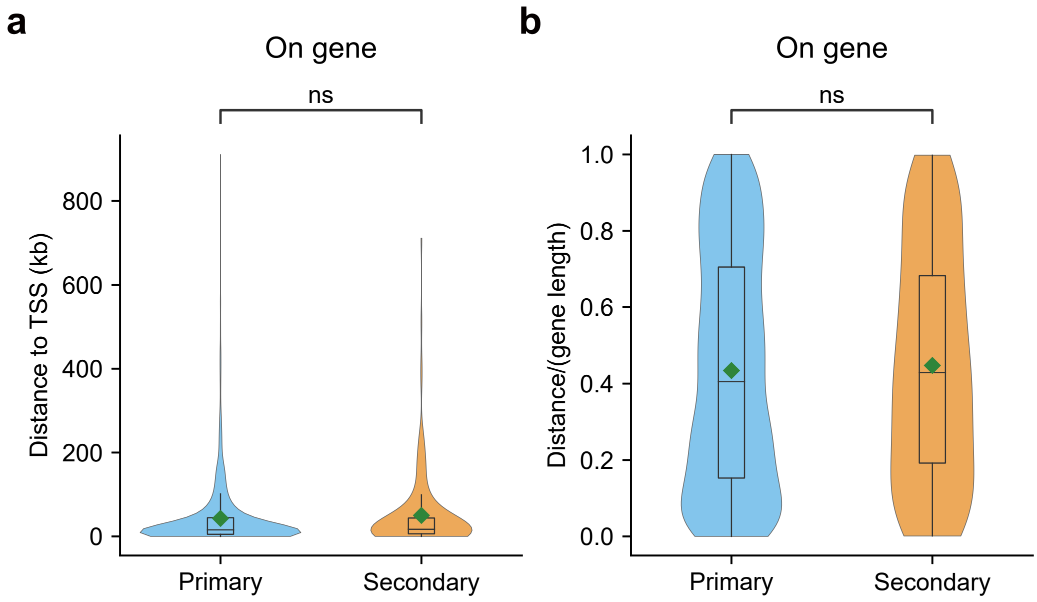

Supplementary Fig. 6. The surrounding 25kb ATAC-seq signal for synovium independent eQTLs (red line) and background SNPs (gray line). Background SNPs were generated from SNPsnap database with matched MAF, LD buddies, distance to nearest gene, and gene density.

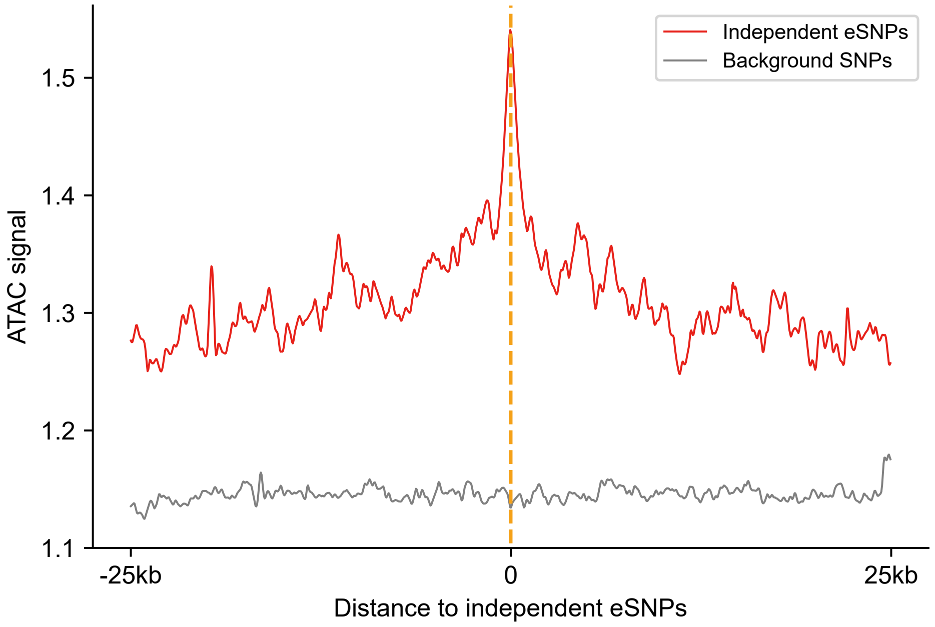

Supplementary Fig. 7. The enrichment of independent SNPs in different histone markers (Supplementary Table 1). The enrichment analysis was performed by Fisher exact test, and the color of heatmap represents the log-transformed fold change (FC) of enrichment. -: $p > 0.05$; *: $p < 0.05$ and adjusted $p > 0.05$; **: adjusted $p < 0.05$. N = 5380, 14311 for independent eQTL SNPs and background SNPs, respectively. Background SNPs were generated from SNPsnap database with matched MAF, LD buddies, distance to nearest gene, and gene density.

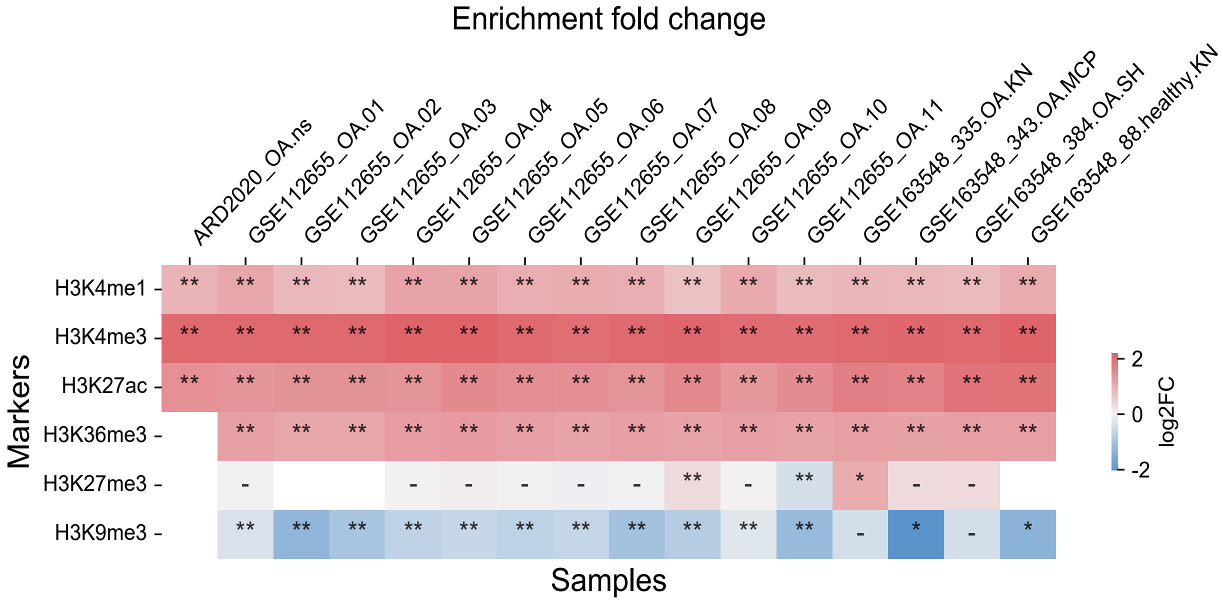

Supplementary Fig. 8. The mashR sharing between our synovium eQTL and GTEx 49 tissues eQTL.

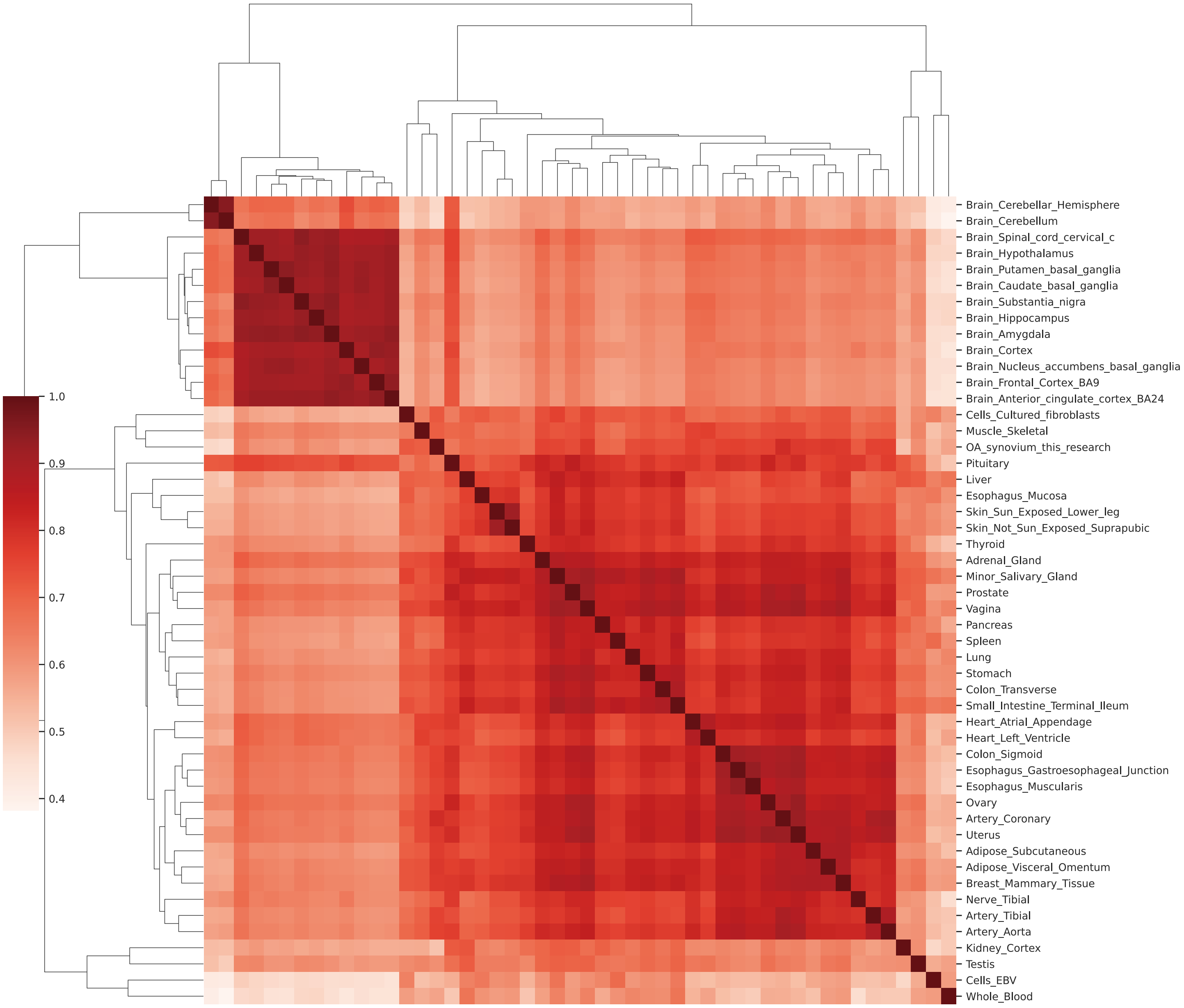

Supplementary Fig. 9. The mashR sharing among our synovium eQTL, previous synovium eQTL, Blueprint immune cells eQTLs, and the DICE immune cell eQTLs.

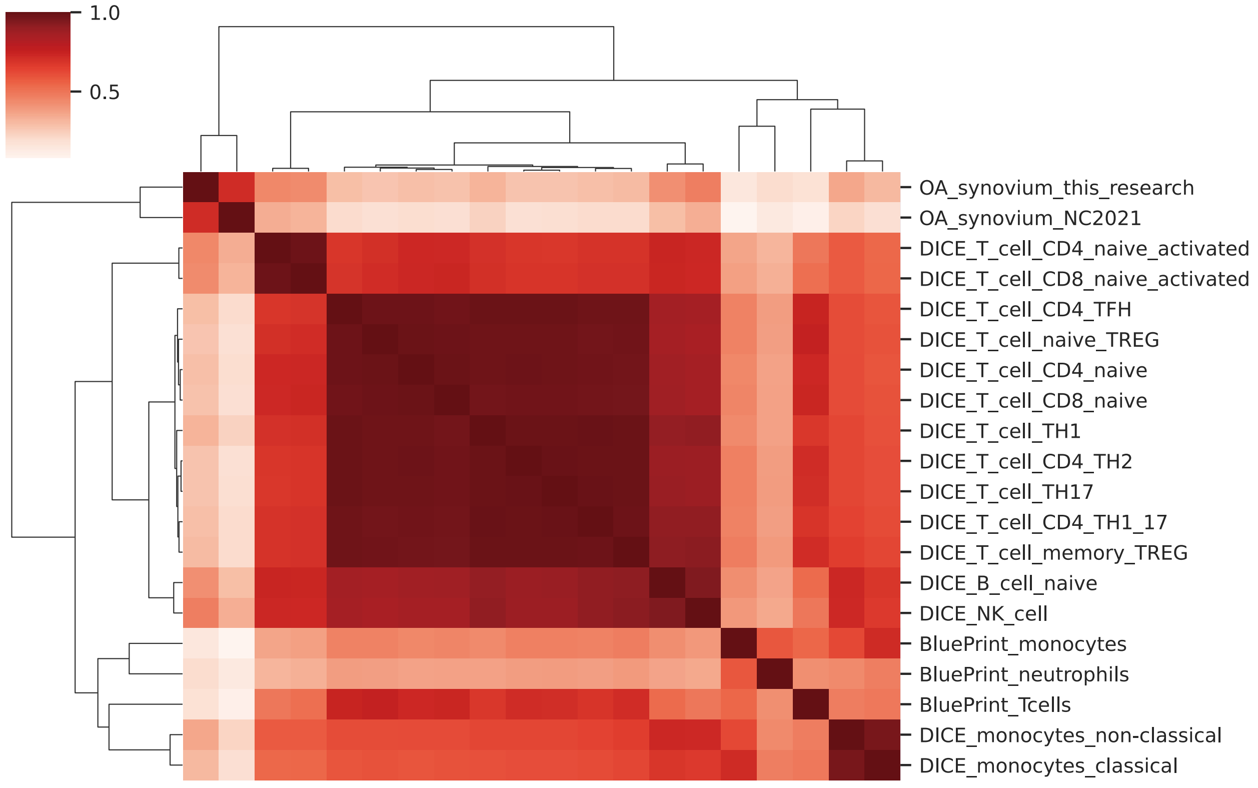

Supplementary Fig. 10. The Protein-Protein interaction (PPI) network for 84 colocalized genes. The hub genes were shown in bold font.

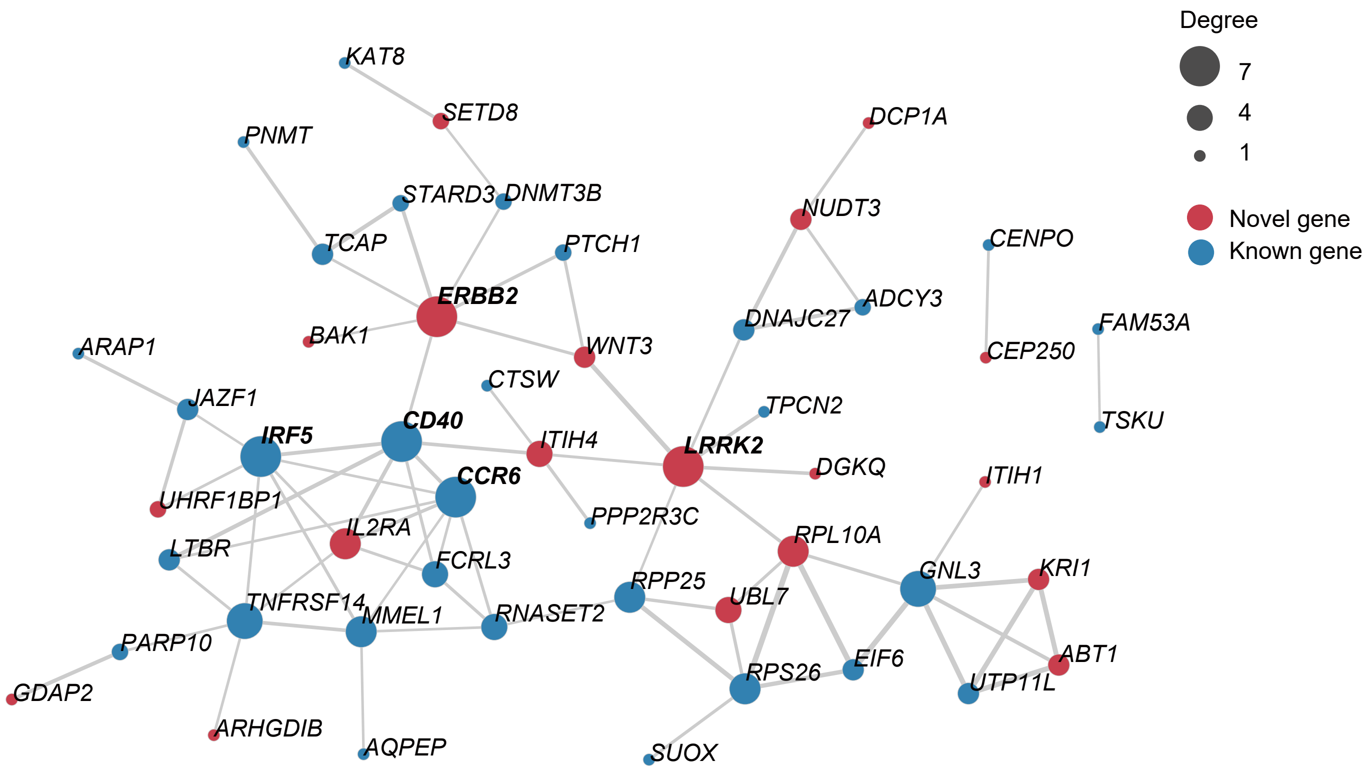

Supplementary Fig. 11. Colocalization locus plots of (a) *IRF5*, (b) *CCR6*, and (c) *CD40*. Red lines represent the corresponding threshold of eQTL or GWAS association *P*-values.

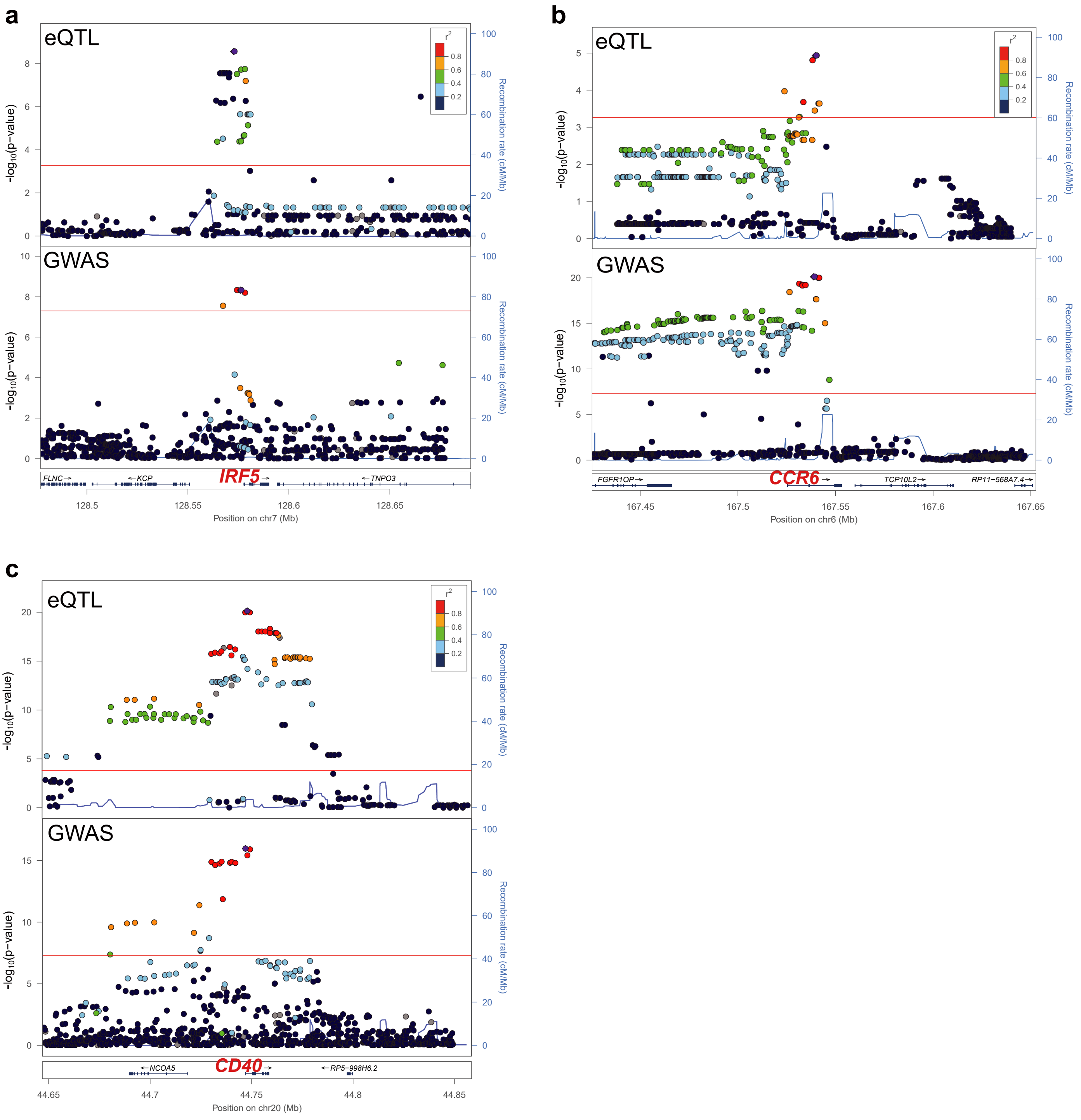

Supplementary Fig. 12. Pathway enrichment analysis for RA colocalized genes. The figure showed the enrichment of fold change and the log-transformed adjusted *P* values (Two-sided hypergeometric tests, adjustments were made for multiple comparisons.). All pathways were enriched with adjusted *P* <0.05. Red dotted lines represent the fold change = 1 and -log10(adj*P*) = 1.31 (corresponding to adjusted *P* = 0.05) respectively.

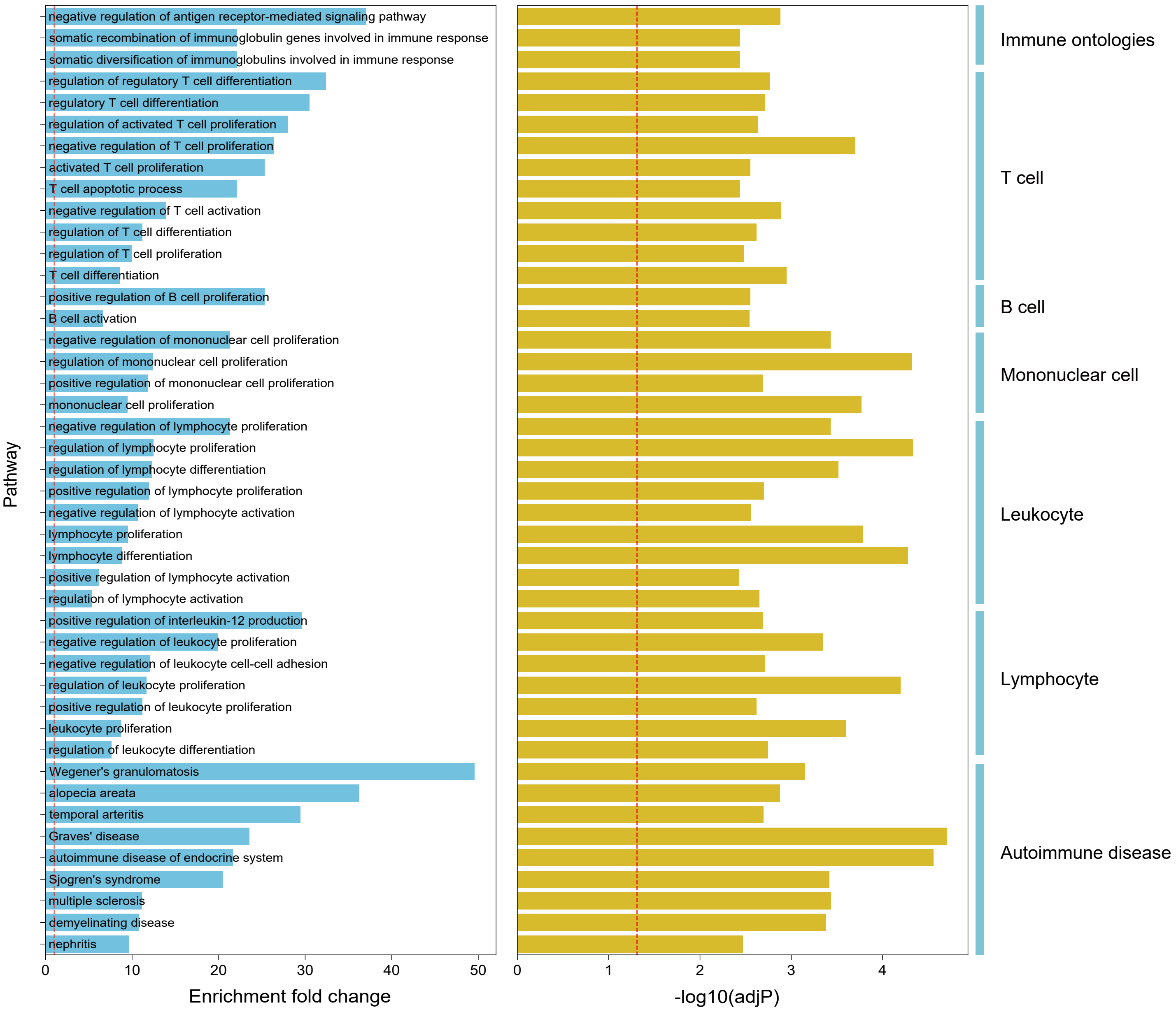

Supplementary Fig. 13. The eQTL boxplot for rs142845557 in *JAZF1*. The boxplots represent 25th, 50th (median), and 75th percentiles, and whiskers extend to 1.5 times the interquartile range, outliers were not shown in the plot. Two-sided t test, P = 7.3E-8. N = 202 individuals.

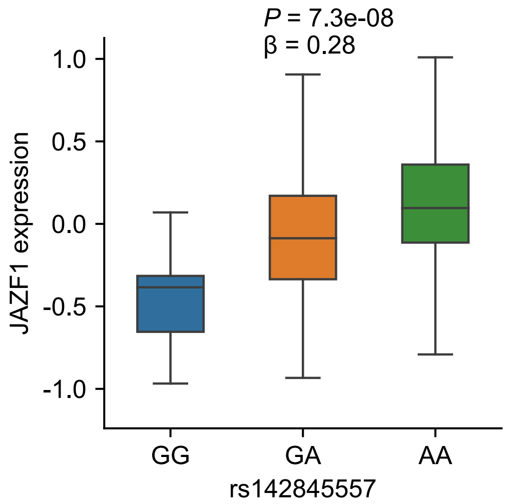

Supplementary Fig. 14. The epigenomic annotation in rs142845557 region.

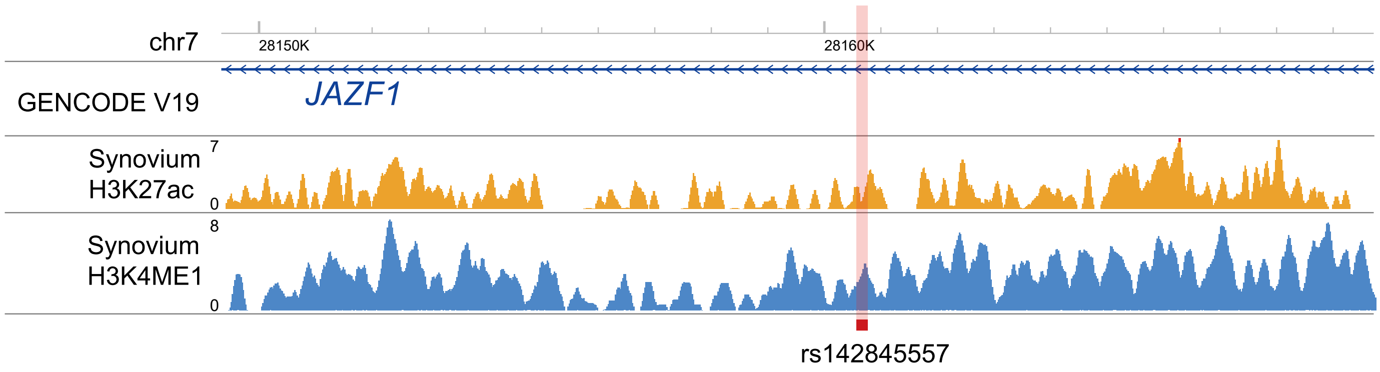

Supplementary Fig. 15. The genotyping and knockout of rs142845557 region. (a) Genotyping of rs142845557 in rheumatoid fibroblast-like synoviocytes (MH7A cells). (b) The scheme for deletion of the region containing SNP rs142845557. We designed one pair of sgRNAs (sgRNA1 and sgRNA2). The original PCR amplified fragment was 918bp, when some fragments were knocked out (358bp), a 560bp fragment would be generated. The gel result of PCR showed the knockout efficiency of rs142845557.

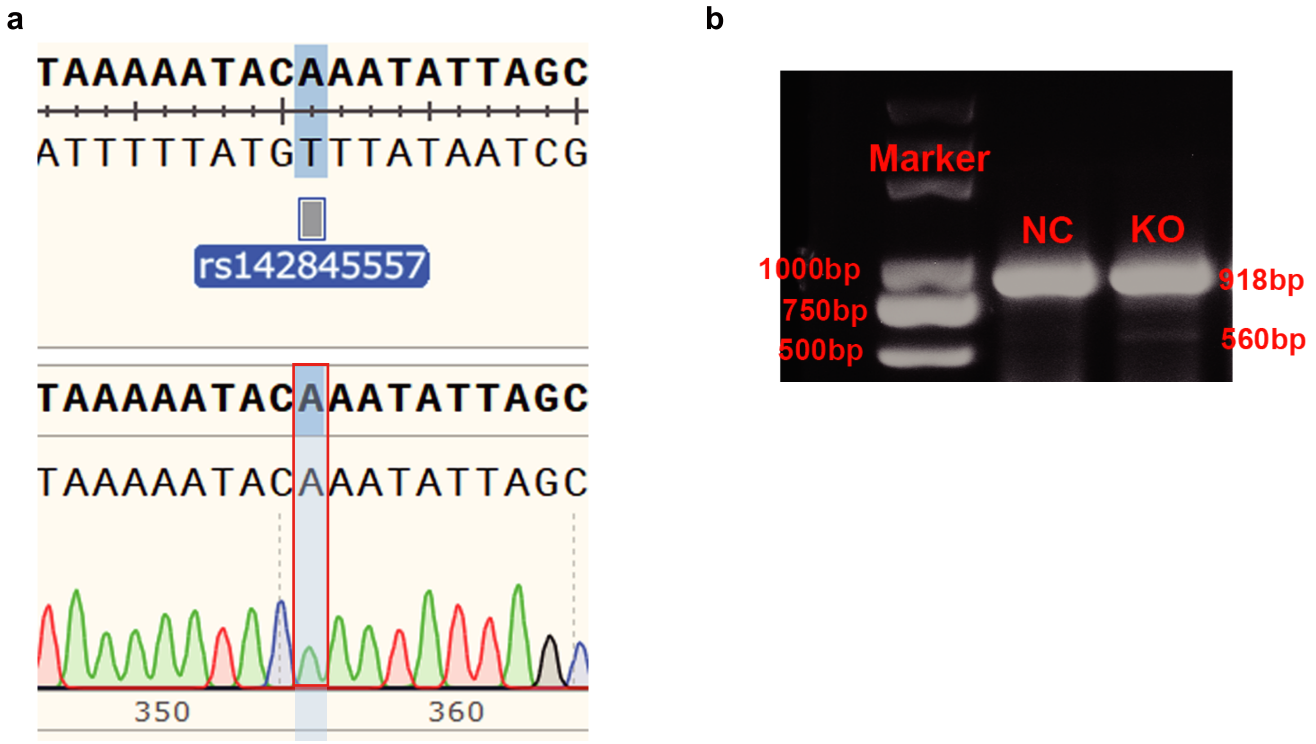

Supplementary Fig. 16. The performance of prediction models under different SVM parameters (t, k, l). (a) AUC under different t and k. (b) AUC under different t and l.

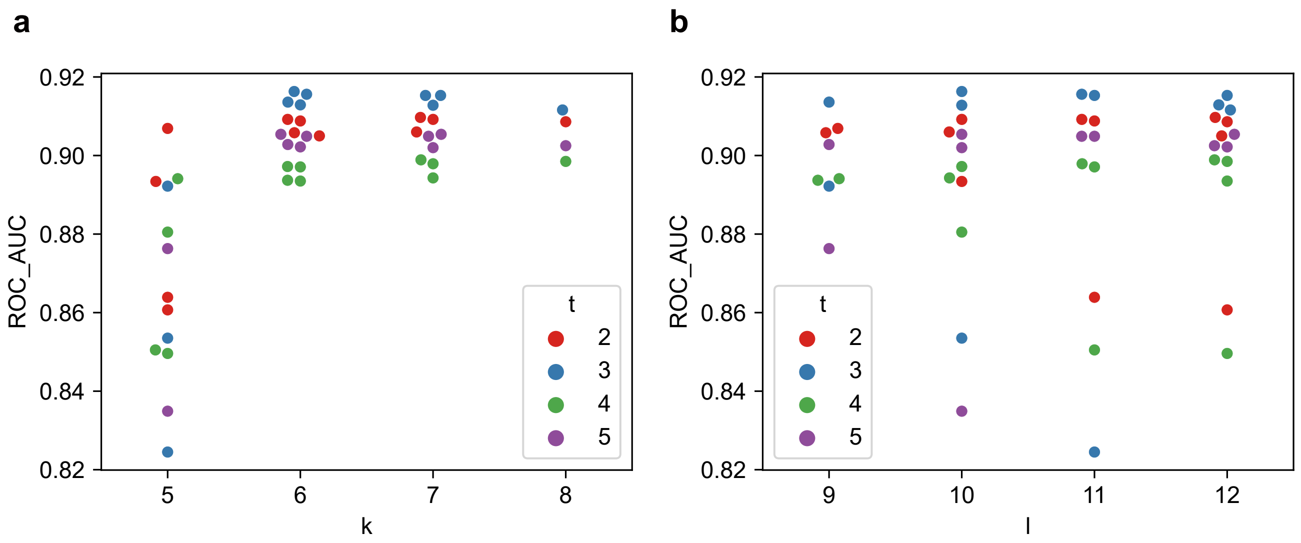

Supplementary Fig. 17. ROC curves of the optimal gkm-SVM model. (a) 3-fold cross validation ROC curves of the optimal model. (b) The independent test ROC curves in 11 synovium ATAC-seq samples[21].

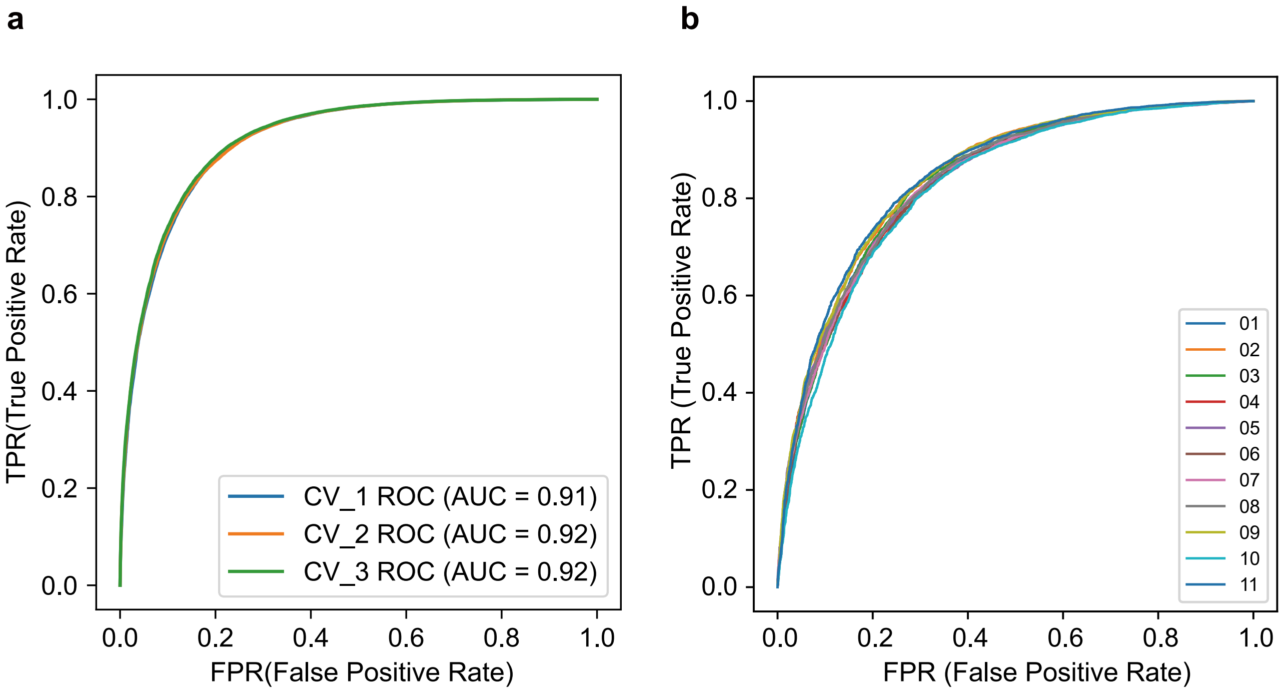

Supplementary Fig. 18. Q-Q plot of eQTac mapping. The x-axis represents expected quantiles under null distribution, and the y-axis represents observed quantiles. Red dashed line represents y = x.

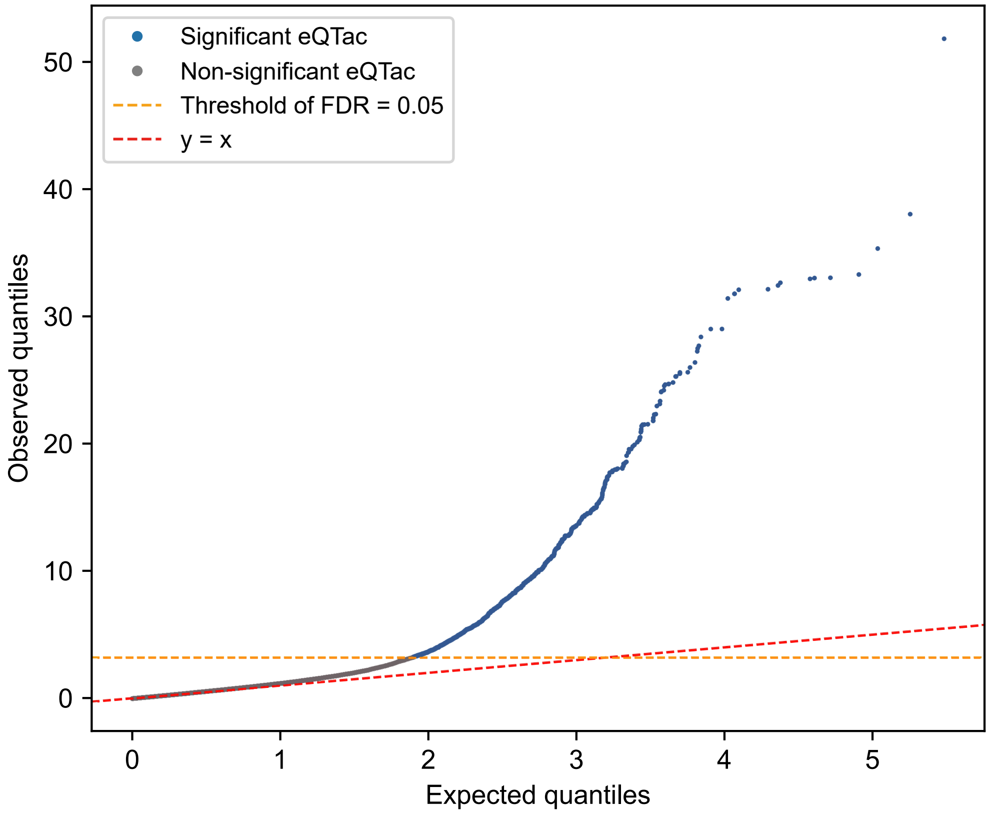

Supplementary Fig. 19. EpiMap chromatin states annotation (HMM18) (Supplementary Table 6) enrichment analysis for eQTac regions. Heatmap showed the enrichment of significant PREs compared with non-significant PREs. Two-sided Fisher exact test. N = 1517 and 8379 for significant PREs and non-significant PREs, respectively.

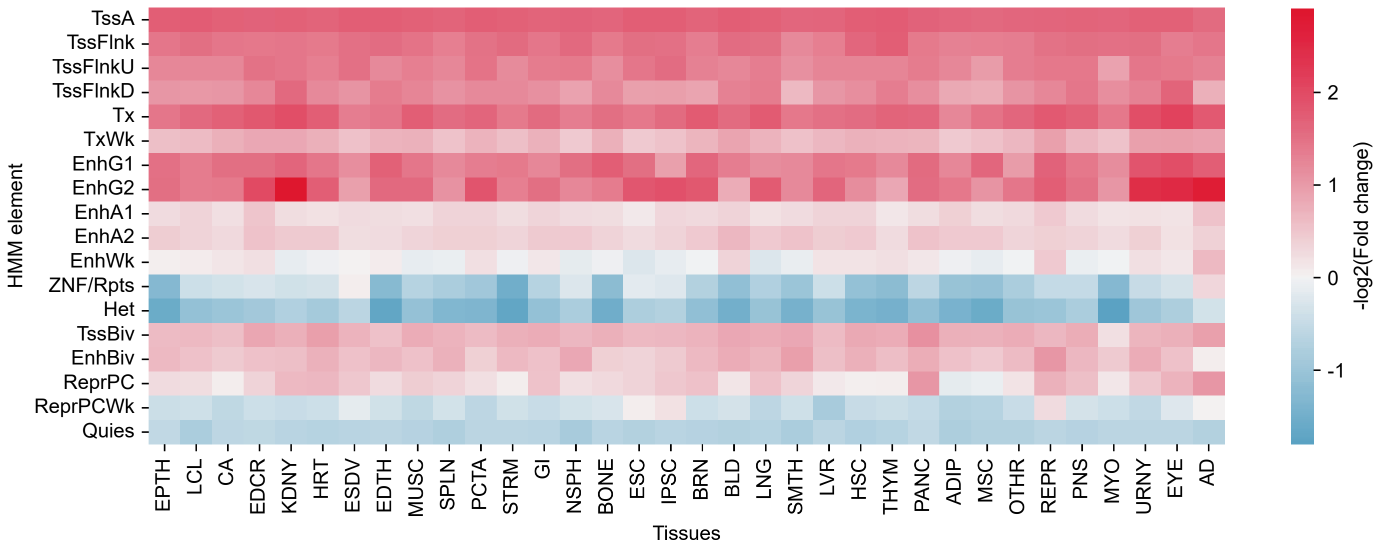

Supplementary Fig. 20. Box plot for four SNPs eQTL with *BAK1* that located on the PRE region chr6:33581434-33581934. The boxplots represent 25th, 50th (median), and 75th percentiles, and whiskers extend to 1.5 times the interquartile range, outliers were not shown in the plot. Two-sided t test. No adjustments were made for multiple comparisons. N = 202 individuals.

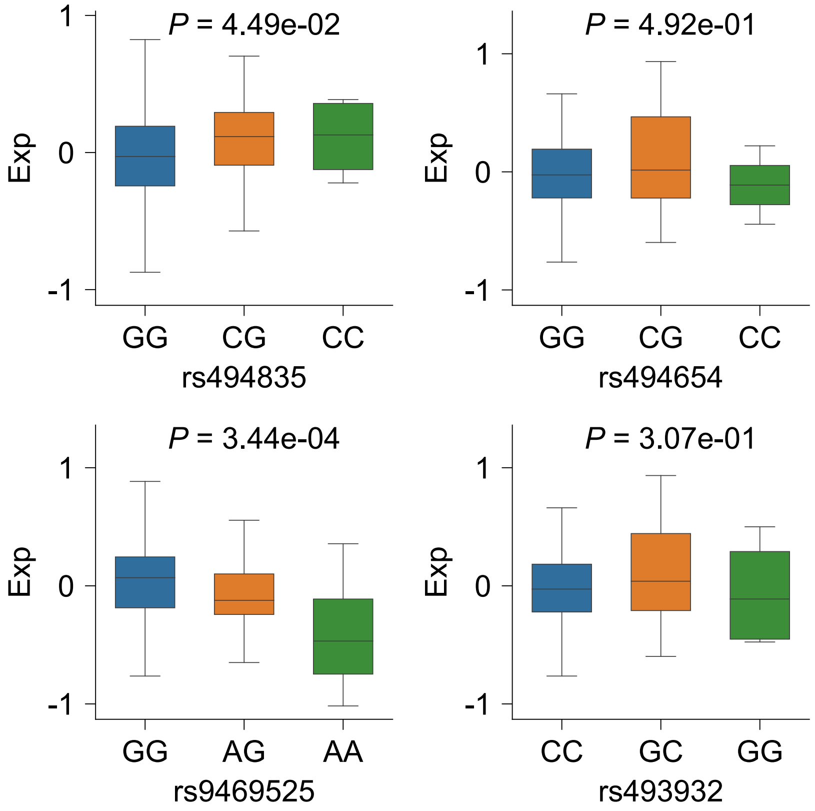

Supplementary References

1. Ge, X., et al., *Functional genomics atlas of synovial fibroblasts defining rheumatoid arthritis heritability.* Genome Biol, 2021. 22(1): p. 247.
2. Ai, R., et al., *Comprehensive epigenetic landscape of rheumatoid arthritis fibroblast-like synoviocytes.* Nat Commun, 2018. 9(1): p. 1921.
3. Tsuchiya, H., et al., *Parsing multiomics landscape of activated synovial fibroblasts highlights drug targets linked to genetic risk of rheumatoid arthritis.* Ann Rheum Dis, 2021. 80(4): p. 440-450.
4. Boer, C.G., et al., *Deciphering osteoarthritis genetics across 826,690 individuals from 9 populations.* Cell, 2021. 184(24): p. 6003-6005.
5. Zengini, E., et al., *Genome-wide analyses using UK Biobank data provide insights into the genetic architecture of osteoarthritis.* Nat Genet, 2018. 50(4): p. 549-558.
6. Tachmazidou, I., et al., *Identification of new therapeutic targets for osteoarthritis through genome-wide analyses of UK Biobank data.* Nat Genet, 2019. 51(2): p. 230-236.
7. Okada, Y., et al., *Genetics of rheumatoid arthritis contributes to biology and drug discovery.* Nature, 2014. 506(7488): p. 376-81.
8. Ishigaki, K., et al., *Large-scale genome-wide association study in a Japanese population identifies novel susceptibility loci across different diseases.* Nat Genet, 2020. 52(7): p. 669-679.
9. Ha, E., S.C. Bae, and K. Kim, *Large-scale meta-analysis across East Asian and European populations updated genetic architecture and variant-driven biology of rheumatoid arthritis, identifying 11 novel susceptibility loci.* Ann Rheum Dis, 2021. 80(5): p. 558-565.
10. Ellinghaus, D., et al., *Analysis of five chronic inflammatory diseases identifies 27 new associations and highlights disease-specific patterns at shared loci.* Nat Genet, 2016. 48(5): p. 510-8.
11. Hinks, A., et al., *Dense genotyping of immune-related disease regions identifies 14 new susceptibility loci for juvenile idiopathic arthritis.* Nat Genet, 2013. 45(6): p. 664-9.
12. Lambert, C., et al., *Gene expression pattern of cells from inflamed and normal areas of osteoarthritis synovial membrane.* Arthritis Rheumatol, 2014. 66(4): p. 960-8.
13. Huber, R., et al., *Identification of intra-group, inter-individual, and gene-specific variances in mRNA expression profiles in the rheumatoid arthritis synovial membrane.* Arthritis Res Ther, 2008. 10(4): p. R98.
14. Woetzel, D., et al., *Identification of rheumatoid arthritis and osteoarthritis patients by transcriptome-based rule set generation.* Arthritis Res Ther, 2014. 16(2): p. R84.
15. Broeren, M.G., et al., *Functional Tissue Analysis Reveals Successful Cryopreservation of Human Osteoarthritic Synovium.* PLoS One, 2016. 11(11): p. e0167076.
16. Thomas, G.P., et al., *Expression profiling in spondyloarthropathy synovial biopsies highlights changes in expression of inflammatory genes in conjunction with tissue remodelling genes.* BMC Musculoskelet Disord, 2013. 14: p. 354.
17. Simonds, M.M., et al., *The culture microenvironment of juvenile idiopathic arthritis synovial fibroblasts is favorable for endochondral bone formation through BMP4 and repressed by chondrocytes.* Pediatr Rheumatol Online J, 2021. 19(1): p. 72.
18. Nasser, J., et al., *Genome-wide enhancer maps link risk variants to disease genes*. Nature, 2021. 593(7858): p. 238-243.
19. Boix, C.A., et al., Regulatory genomic circuitry of human disease loci by integrative epigenomics. Nature, 2021. 590(7845): p. 300-307.
20. Steinberg, J., et al., *A molecular quantitative trait locus map for osteoarthritis*. Nat Commun, 2021. 12(1): p. 1309.
21. Ai, R., et al., *Comprehensive epigenetic landscape of rheumatoid arthritis fibroblast-like synoviocytes*. Nat Commun, 2018. 9(1): p. 1921.
