# Supplementary material for: A landscape of gene expression regulation for synovium in arthritis
Source: Nat Commun. 2024 Feb 15;15:1409. doi: 10.1038/s41467-024-45652-x (PMC10869817; doi:10.1038/s41467-024-45652-x)
Supplement: Supplementary file 3 — Description of Additional Supplementary Files [file 41467_2024_45652_MOESM3_ESM.pdf]

File Name: Supplementary Data 1
Description: Independent SNPs identified by step-wised conditional regression.

File Name: Supplementary Data 2
Description: GWAS summary data in colocalization and LD score regression analysis.

File Name: Supplementary Data 3
Description: Colocalized genes and related functions of GWAS traits.

File Name: Supplementary Data 4
Description: Gene ontology (GO) and disease ontology (DO) enrichment analysis of genes colocalized with RA.

File Name: Supplementary Data 5
Description: Significant eQTac results with FDR<0.05.
