# Supplementary material for: A landscape of gene expression regulation for synovium in arthritis
Source: Nat Commun. 2024 Feb 15;15:1409. doi: 10.1038/s41467-024-45652-x (PMC10869817; doi:10.1038/s41467-024-45652-x)
Supplement: Supplementary file 9 — Reporting Summary [file 41467_2024_45652_MOESM9_ESM.pdf]

nature portfolio

Corresponding author(s): Tie-Lin Yang
Last updated by author(s): Dec 4, 2023

Reporting Summary

Nature Portfolio wishes to improve the reproducibility of the work that we publish. This form provides structure for consistency and transparency in reporting. For further information on Nature Portfolio policies, see our Editorial Policies and the Editorial Policy Checklist.

Statistics

For all statistical analyses, confirm that the following items are present in the figure legend, table legend, main text, or Methods section.

n/a	Confirmed	
☐	☒	The exact sample size (*n*) for each experimental group/condition, given as a discrete number and unit of measurement
☐	☒	A statement on whether measurements were taken from distinct samples or whether the same sample was measured repeatedly
☐	☒	The statistical test(s) used AND whether they are one- or two-sided<br>*Only common tests should be described solely by name; describe more complex techniques in the Methods section.*
☐	☒	A description of all covariates tested
☐	☒	A description of any assumptions or corrections, such as tests of normality and adjustment for multiple comparisons
☐	☒	A full description of the statistical parameters including central tendency (e.g. means) or other basic estimates (e.g. regression coefficient) AND variation (e.g. standard deviation) or associated estimates of uncertainty (e.g. confidence intervals)
☐	☒	For null hypothesis testing, the test statistic (e.g. *F*, *t*, *r*) with confidence intervals, effect sizes, degrees of freedom and *P* value noted<br>*Give P values as exact values whenever suitable.*
☒	☐	For Bayesian analysis, information on the choice of priors and Markov chain Monte Carlo settings
☒	☐	For hierarchical and complex designs, identification of the appropriate level for tests and full reporting of outcomes
☐	☒	Estimates of effect sizes (e.g. Cohen's *d*, Pearson's *r*), indicating how they were calculated

Our web collection on statistics for biologists contains articles on many of the points above.

Software and code

Policy information about availability of computer code

Data collection	eQTLQC<br>Impute2 v2.3.2_x86_64_static<br>Fastp v0.19.7<br>FastQC v0.11.9<br>STAR v2.7.9a<br>RNA-SeQC v2.3.5<br>SAMtools v1.9<br>QualiMap v.2.2.2-dev<br>Bowtie2 v2.3.5.1<br>MACS2 v2.2.7.1
Data analysis	plink v1.90<br>plink 2.0<br>EIGENSOFT v8.0.0<br>GTEx modified version of FastQTL (https://github.com/francois-a/fastqtl)<br>ANNOVAR version 2020 Jun 08<br>tensorQTL v1.0.6<br>Dap-g v1.0.0<br>LDSC v1.0.1<br>coloc v5.2.0<br>lsgkm v0.1.1

gkmSVM 0.8.0
GCTA v1.94.1
Metasoft v2.0.0
mashR v0.2.73
eQTac v1.0.12 (https://github.com/JFF1594032292/eQTac)

For manuscripts utilizing custom algorithms or software that are central to the research but not yet described in published literature, software must be made available to editors and reviewers. We strongly encourage code deposition in a community repository (e.g. GitHub). See the Nature Portfolio guidelines for submitting code & software for further information.

Data

Policy information about availability of data

All manuscripts must include a data availability statement. This statement should provide the following information, where applicable:

- Accession codes, unique identifiers, or web links for publicly available datasets
- A description of any restrictions on data availability
- For clinical datasets or third party data, please ensure that the statement adheres to our policy

All relevant data support the key findings of this study are available within the article and its Supplementary Information files. The RNA sequencing data and ATAC-seq data generated in this study have been deposited in the Genome Sequence Archive in National Genomics Data Center, China National Center for Bioinformation / Beijing Institute of Genomics, Chinese Academy of Sciences under accession code HRA004624 (https://ngdc.cncb.ac.cn/gsa-human/browse/HRA004624), which are available under restricted access for privacy protection. Access can be obtained by reasonable request. The raw genotype data are protected and are not available due to data privacy laws. The processed independent eQTLs, colocalization results, and significant eQTac data are available at Supplementary Data. Other public data used in the study are listed in Supplementary Tables and Supplementary Data.

Datasets used in epigenetic annotation for independent SNPs were available in
https://www.ncbi.nlm.nih.gov/geo/download/?acc=GSE163548&format=file
https://www.ncbi.nlm.nih.gov/geo/query/acc.cgi?acc=GSE112655
https://humandbs.biosciencedbc.jp/files/hum0207/hum0207.v1.ChIP.v1.zip

20 osteoarthritis GWAS summary data were available in
https://msk.hugeamp.org/downloads.html
http://ftp.ebi.ac.uk/pub/databases/gwas/summary_statistics/GCST005001-GCST006000/GCST005811/harmonised/29559693-GCST005811-EFO_0002506-build37.f.tsv.gz
http://ftp.ebi.ac.uk/pub/databases/gwas/summary_statistics/GCST005001-GCST006000/GCST005812/harmonised/29559693-GCST005812-EFO_0002506-build37.f.tsv.gz
http://ftp.ebi.ac.uk/pub/databases/gwas/summary_statistics/GCST005001-GCST006000/GCST005813/harmonised/29559693-GCST005813-EFO_0004616-build37.f.tsv.gz
http://ftp.ebi.ac.uk/pub/databases/gwas/summary_statistics/GCST005001-GCST006000/GCST005814/harmonised/29559693-GCST005814-EFO_0002506-Build37.f.tsv.gz
http://ftp.ebi.ac.uk/pub/databases/gwas/summary_statistics/GCST005001-GCST006000/GCST005810/harmonised/29559693-GCST005810-EFO_1000786-build37.f.tsv.gz
http://ftp.ebi.ac.uk/pub/databases/gwas/summary_statistics/GCST007001-GCST008000/GCST007090/Tachmazidou_30664745_KNEEOA.txt.gz
http://ftp.ebi.ac.uk/pub/databases/gwas/summary_statistics/GCST007001-GCST008000/GCST007091/Tachmazidou_30664745_HIPOA.txt.gz
http://ftp.ebi.ac.uk/pub/databases/gwas/summary_statistics/GCST007001-GCST008000/GCST007092/Tachmazidou_30664745_HIPKNEEOA.txt.gz
http://ftp.ebi.ac.uk/pub/databases/gwas/summary_statistics/GCST007001-GCST008000/GCST007093/Tachmazidou_30664745_ALLOA.txt.gz

5 rheumatoid arthritis GWAS summary data were available in
http://jenger.riken.jp:8080/pheno/Rheumatoid_Arthritis_Asian
http://jenger.riken.jp:8080/pheno/Rheumatoid_Arthritis_European
http://jenger.riken.jp:8080/pheno/Rheumatoid_Arthritis_TransEthnic
http://jenger.riken.jp/93pheweb_gwas/
http://ftp.ebi.ac.uk/pub/databases/gwas/summary_statistics/GCST90013001-GCST90014000/GCST90013534/harmonised/33310728-GCST90013534-EFO_0000685-Build37.f.tsv.gz

8 mental disorder GWAS summary data were available in
https://pgc.unc.edu/for-researchers/download-results/
https://www.ncbi.nlm.nih.gov/projects/gap/cgi-bin/study.cgi?study_id=phs001672.v1.p1

The predicted enhancer-gene interaction from ABC model were available in ftp://ftp.broadinstitute.org/outgoing/lincRNA/ABC/AllPredictions.AvgHiC.ABC0.015.minus150.ForABCPaperV3.txt.gz

The predicted enhancer-gene interaction from EpiMap were available in https://personal.broadinstitute.org/cboix/epimap/links/pergroup/

The HMM18 state model from EpiMap were available in https://personal.broadinstitute.org/cboix/epimap/ChromHMM/observed_aux_18_hg19/CALLS/

The called loops data from synovium were available in https://humandbs.biosciencedbc.jp/files/hum0207/hum0207.v1.HiC.v1.zip

The capture Hi-C from synovium were available in
https://ftp.ncbi.nlm.nih.gov/geo/series/GSE163nnn/GSE163548/suppl/GSE163548_Stim_Chicago_washU_text.txt.gz
https://ftp.ncbi.nlm.nih.gov/geo/series/GSE163nnn/GSE163548/suppl/GSE163548_Unstim_Chicago_washU_text.txt.gz

The GEO datasets utilized in differential expression analysis were available in accession number GSE12021, GSE165626, GSE41038, GSE46750, GSE55235, GSE55457, GSE82107

The independent validation datasets with 92 individuals genotype,RNAseq, and ATACseq data were applied from dbGAP(phs000815).

Research involving human participants, their data, or biological material

Policy information about studies with human participants or human data. See also policy information about sex, gender (identity/presentation), and sexual orientation and race, ethnicity and racism.

Reporting on sex and gender	Our study contained 77 men and 168 women, gender information were from the self-report. Then we checked the consistence of genotype-derived sex and self-reported gender, and removed the inconsistent individuals. The sex information were used as covariates in the eQTL and eQTac analysis.
Reporting on race, ethnicity, or other socially relevant groupings	All participants were Chinese Han adults, the ethnic information were from the self-report.
Population characteristics	Osteoarthritis patients undergoing knee joint replacement surgery (77 men, 168 women, age 46-84 years, mean 67 years), with no history of significant knee surgery, infection, or fracture, and no malignancy within the previous 5 years.
Recruitment	All participants were recruited from the open recruitment of clinical research.<br>Participation in this study was voluntary and may have led to self-selection bias.<br>The age, sex and the past medical history may cause bias in the association analysis. To minimize the impact of these factors, we have excluded the individuals with history of significant knee surgery, infection, or fracture, and no malignancy within the previous 5 years. We also included the sex and age as covariates in all association analysis.
Ethics oversight	The study was approved by the Ethics Committee of Xi'an Jiaotong University Honghui Hospital. All patients were provided written, informed consent before participating in the study.

Note that full information on the approval of the study protocol must also be provided in the manuscript.

Field-specific reporting

Please select the one below that is the best fit for your research. If you are not sure, read the appropriate sections before making your selection.

☒ Life sciences ☐ Behavioural & social sciences ☐ Ecological, evolutionary & environmental sciences

For a reference copy of the document with all sections, see nature.com/documents/nr-reporting-summary-flat.pdf

Life sciences study design

All studies must disclose on these points even when the disclosure is negative.

Sample size	Sample sizes before QC exclusion: RNA-seq: 210, Genotype: 245, ATAC-seq: 10. No statistical power calculations were used to determine the same size. Sample sizes was determined by the availability and quality of the human samples.
Data exclusions	Genotype data: 2 patients were excluded because of high heterozygosity rate deviated more than 3 standard deviations from the heterozygosity rate mean.<br>RNA data: 6 synovial samples were excluded because of the low pairwise expression correlation coefficients with other samples.
Replication	We compared our eQTl results and previous published synovial eQTL results and 22157/590217 SNP-gene pairs could be replicated. The low replicated ratio was caused by the different population and much larger sample size of our research. The intersect significant eQTLs showed the concordance of pearson r=0.84, and >95.5% eQTLs showed the same direction.
Randomization	not applicable due to no experimental treatments
Blinding	All samples in this study were collected from osteoarthritis patients. Samples from each tissue were analyzed separately to identify molecular QTL analysis, so blinding was not applicable. Investigators were blind in the fragment deletion experiments.

Reporting for specific materials, systems and methods

We require information from authors about some types of materials, experimental systems and methods used in many studies. Here, indicate whether each material, system or method listed is relevant to your study. If you are not sure if a list item applies to your research, read the appropriate section before selecting a response.

Materials & experimental systems

n/a	Involved in the study
☒	☐ Antibodies
☐	☒ Eukaryotic cell lines
☒	☐ Palaeontology and archaeology
☒	☐ Animals and other organisms
☒	☐ Clinical data
☒	☐ Dual use research of concern
☒	☐ Plants

Methods

n/a	Involved in the study
☒	☐ ChIP-seq
☒	☐ Flow cytometry
☒	☐ MRI-based neuroimaging

Eukaryotic cell lines

Policy information about cell lines and Sex and Gender in Research

Cell line source(s)	MH7A cell lines were obtained from Shanghai Guan&Dao Biological Engineering Co., Ltd.
Authentication	authenticated using short tandem repeat (STR) profiling by scientific service at Beijing Tsingke Biotech (Beijing, China).
Mycoplasma contamination	Mycoplasma tests are performed on a regular basis.
Commonly misidentified lines (See ICLAC register)	No commonly misidentified cell lines were used.
